# Comprehensive LC-ESI-HRMS/MS Profiling and Assessment of Texture, Predicted Glycaemic Index, Antioxidant Activity and Digestive Enzyme Inhibition of Gluten- and Lactose-Free Cookies Enriched with Pomegranate By-Products

**DOI:** 10.3390/foods15030457

**Published:** 2026-01-28

**Authors:** Roberta Pino, Rosa Tundis, Vincenzo Sicari, Antonio Mincione, Antonio Gattuso, Chiara La Torre, Alessia Fazio, Sonia Piacente, Milena Masullo, Antonietta Cerulli, Monica Rosa Loizzo

**Affiliations:** 1 Department of Pharmacy, Health and Nutritional Sciences, University of Calabria, 87036 Rende, CS, Italy; roberta.pino@unical.it (R.P.); rosa.tundis@unical.it (R.T.); chiara.latorre@unical.it (C.L.T.); a.fazio@unical.it (A.F.); monica_rosa.loizzo@unical.it (M.R.L.); 2 Department of Agraria, Mediterranea University of Reggio Calabria, Cittadella Universitaria, Località Feo di Vito, 89124 Reggio Calabria, RC, Italy; amincione@unirc.it (A.M.); antonio.gattuso@unirc.it (A.G.); 3 Department of Pharmacy, University of Salerno, 84084 Fisciano, SA, Italy; piacente@unisa.it (S.P.); mmasullo@unisa.it (M.M.);

**Keywords:** pomegranate seeds, by-product valorisation, simultaneously gluten- and lactose-free cookies, glycaemic index, functional foods

## Abstract

This study investigated gluten- and lactose-free cookies enriched with pomegranate seed flour (PSF, 5 and 10% *w*/*w*), a sustainable by-product of juice processing. LC-ESI/HRMS/MS analysis of PSF identified 36 bioactive compounds, mainly flavonoids, phenolic acids, hydrolysable tannins, and polar lipids. PSF incorporation significantly affected colour and texture, increasing friability, as evidenced by a reduction in breaking force from 35.37 N in the control cookie to 21.72 N in cookies enriched with 10% PSF, while maintaining good sensory acceptability. Total phenol (≈1.60–1.82 mg GAE/g) and flavonoid contents were only slightly affected by PSF addition; however, antioxidant activity markedly increased, with FRAP values rising from 55.8 to 67.82 μM Fe (II)/g and DPPH IC_50_ values decreasing from 31.38 to 12.72 μg/mL in the 10% PSF-enriched cookies. The enriched cookies inhibited pancreatic lipase, α-amylase, and α-glucosidase in a clear concentration-dependent manner and showed a reduced predicted glycaemic index (pGI 46.80 vs. 50.08 in the control). Multivariate analysis confirmed a clear dose-dependent effect of PSF on functional, textural, and sensory properties. Overall, pomegranate seed flour proved to be an effective upcycled ingredient for enhancing the functional profile of gluten- and lactose-free bakery products. Further studies using digestion models and in vivo or clinical approaches are needed to clarify the nutritional relevance and health effects of PSF-enriched foods.

## 1. Introduction

In recent years, the demand for bakery goods designed for consumers with specific nutritional needs, such as individuals with celiac disease and lactose intolerance, has steadily increased. Celiac disease is an autoimmune disorder affecting the small intestine, impairing nutrient absorption and requiring a lifelong gluten-free diet as the only effective treatment [1]. Accordingly, the global gluten-free market is experiencing substantial growth, with an estimated annual increase of approximately 9.8% until 2030 [2].

Similarly, lactose intolerance, caused by lactase deficiency and associated with gastrointestinal symptoms including abdominal pain, bloating, and diarrhoea [3], is driving the expansion of the lactose-free food sector, which is projected to grow at an annual rate of about 8% by 2031 [4]. In parallel, metabolic syndrome represents a major global public health concern, affecting approximately 32% of the worldwide population [5]. Metabolic syndrome is characterized by a cluster of interrelated conditions, such as obesity, insulin resistance, dyslipidaemia, oxidative stress, and chronic low-grade inflammation, which collectively compromise metabolic health.

Recent evidence indicates that individuals with celiac disease have an increased risk of developing metabolic syndrome and non-alcoholic fatty liver disease, both associated with elevated cardiovascular risk [6]. This vulnerability may be further aggravated by gluten-free diets, which are often nutritionally unbalanced, being rich in simple sugars and fats but poor in dietary fibre and essential micronutrients. Moreover, celiac patients frequently present concomitant lactose intolerance, leading to reduced consumption of milk and dairy products [7] and potentially inadequate calcium and vitamin D intake, with negative implications for bone and overall health.

In this context, the reuse of food industry by-products has gained increasing attention as a strategy aligned with circular economy principles aimed at waste reduction and resource valorisation [8]. Fruit processing by-products represent a valuable source of functional ingredients for food applications [9]. Among these, pomegranate (*Punica granatum* L.) has been widely studied for its health-promoting properties [10].

Pomegranate juice is characterized primarily by soluble sugars (e.g., citric and malic acids) such as glucose and fructose, organic acids, and water-soluble polyphenols (mostly ellagic acid, punicalin and punicalagin [11,12,13].

Pomegranate seeds account for approximately 20% of the fruit weight and constitute the main by-product of juice production, with more than 100,000 tons discarded annually [14]. These by-products contain significant amounts of polyunsaturated fatty acids (notably punicic acid), proteins, phenolic compounds (e.g., ellagitannins), sterols, tocopherols, and dietary fibre [15,16,17,18,19], each of which exhibit antioxidant, anti-inflammatory, and prebiotic activities [20,21]. Moreover, bioactive compounds from agri-food by-products, such as pomegranate polyphenols, have been shown to positively modulate oxidative stress, insulin sensitivity, lipid metabolism, and inflammatory pathways, thereby supporting body weight regulation [22].

Their conversion into flour represents an effective approach to promote sustainability while enhancing the nutraceutical value of food products. Incorporating this ingredient into bakery formulations is consistent with current guidelines for sustainable food production, meeting consumer expectations for health-oriented and environmentally responsible products [23].

Sweet bakery products, such as cookies, are among the most widely consumed foods worldwide, commonly eaten as snacks or breakfast items [24].

Although pomegranate-derived ingredients have been sporadically investigated in bakery products, this study addresses a combination of aspects that has not been previously reported in the literature. To the best of our knowledge, this is the first study to develop gluten-free and lactose-free cookies that are simultaneously enriched with a pomegranate seed by-product (PSF) derived from juice processing, explicitly targeting both nutritional functionality and by-product valorisation within a sustainable food design framework.

A further and substantial element of novelty is the comprehensive LC-ESI-HRMS/MS characterization of PSF, which extends beyond phenolic compounds to include polar lipid profiling, a component that remains largely unexplored in bakery and gluten-free formulations. Importantly, this chemical characterization is directly linked to product formulation and biological functionality, strengthening the translational relevance of the findings.

Moreover, the study is distinguished by a fully integrated and systematic evaluation of technological, nutritional, and functional properties within a single formulation study, rather than addressing these aspects separately, as commonly reported. This includes instrumental texture and colour analysis, dietary fibre enrichment, antioxidant capacity assessed through multiple complementary in vitro assays, predicted glycaemic index, and the inhibition of key digestive enzymes associated with metabolic syndrome and obesity. Such a multidimensional approach to gluten-free bakery product development using a fruit-derived by-product has not been previously documented.

Within this framework, gluten- and lactose-free cookies were formulated with PSF at two inclusion levels (5 and 10% *w*/*w*) and evaluated in vitro for physicochemical properties, dietary fibre content, total phenol and flavonoid contents, and antioxidant activity (ABTS, FRAP, DPPH, and β-carotene bleaching assays). In addition, their bioactive potential related to metabolic syndrome and obesity was assessed through α-amylase, α-glucosidase, and pancreatic lipase inhibition, while consumer-oriented attributes, including colour and texture, were analysed to support product acceptability.

## 2. Materials and Methods

### 2.1. Chemicals and Reagents

All solvents employed in this study were supplied by VWR International S.r.l. (Milan, Italy). Amyloglucosidase and glucose oxidase/peroxidase assay kit (GOPOD format) were purchased from Megazyme, Wicklow, Ireland. Gallic acid, caffeic acid, chlorogenic acid, *p*-coumaric acid, ferulic acid, ellagic acid, quercetin, catechin, rutin, ascorbic acid, propyl gallate, butylated hydroxytoluene (BHT), β-carotene, linoleic acid, Tween 20, sodium potassium tartrate, sodium chloride, sodium carbonate, ferric chloride (FeCl_3_), Folin-Ciocalteu reagent, 2,2-diphenyl-1-picrylhydrazyl (DPPH), 2,4,6-tripyridyl-s-triazine (TPTZ), 2,2′-azino-bis(3-ethylbenzothiazoline-6-sulfonic acid) diammonium salt (ABTS), phosphate buffer, potato starch, α-amylase enzyme, α-glucosidase enzyme, dihydrochloride, maltose, potassium chlorate, aluminium chloride, sodium acetate, *o*-dianisidine (DIAN) solution, peroxidase/glucose oxidase (PGO) colorimetric reagent, and dimethyl sulfoxide (DMSO) were purchased from Sigma-Aldrich S.r.l. (Milan, Italy).

### 2.2. Pomegranate By-Products (PSF) Preparation and Extraction Procedure

The exhausted seeds, obtained as a by-product of the pomegranate juice extraction process, were first separated from the liquid fraction and subjected to air-dried, followed by oven drying at 60 °C for 24 h (Heraeus Series 6000, Thermo Fisher Scientific, Milan, Italy) at controlled temperature (~45 °C) until constant weight was achieved, in order to preserve thermolabile bioactive compounds and prevent oxidative degradation. At the end of the process, the dried seeds were allowed to cool to room temperature and then ground using IKA A 10 basic laboratory milling apparatus (IKA-Werke GmbH & Co. KG, Staufen, Germany). The obtained flour was subsequently sieved to standardize the particle size below 0.5 mm, resulting in a fine and homogeneous powder flour (PSF).

PSF was subjected to ultrasound-assisted extraction using a hydroalcoholic solvent (ethanol/water, 9:1 *v*/*v*) at a 1:1 sample-to-solvent *ratio*. Sonication was performed in three 30 min cycles at 40 kHz and 40 °C using a thermostatic ultrasonic bath. After filtration through a 0.45 μm membrane, solvents were removed and the PSF extract was stored at 4 °C prior to analysis [25].

### 2.3. LC-ESI/HRMS/MS Analysis of Pomegranate By-Products (PSF)

Qualitative UHPLC–(−)ESI–HRMS/MS analysis was carried out using an UltiMate 3000 UHPLC system (Thermo Fisher Scientific, Waltham, MA, USA) coupled to a Q Exactive mass spectrometer (Thermo Fisher Scientific, Waltham, MA, USA). The PSF extract (1.0 mg/mL in methanol/water, 1:1, *v*/*v*) was injected (6 μL) for analysis.

Separation was achieved on a Luna C18 column (150 × 2.1 mm, 5 μm) at 0.2 mL/min using water and acetonitrile (both with 0.1% formic acid) under gradient elution (5–95% B). Full-scan MS data were acquired over an *m*/*z* range of 150–1500, with MS/MS performed in data-dependent mode at a collision energy of 30%.

ESI source parameters were set to −48 V (capillary), −176.47 V (tube lens), and 280 °C (source temperature). Instrument calibration, data acquisition, and processing were performed using Xcalibur 2.2 software (Thermo Fisher Scientific, Waltham, USA) [26].

### 2.4. Cookies Making Procedure

For the preparation of the three cookie formulations, B (control sample without PSF enrichment), B5 (enriched with 5% pomegranate seed flour (PSF)), and B10 (enriched with 10% PSF) the ingredients listed in Table 1 were used.

**Figure 1:**
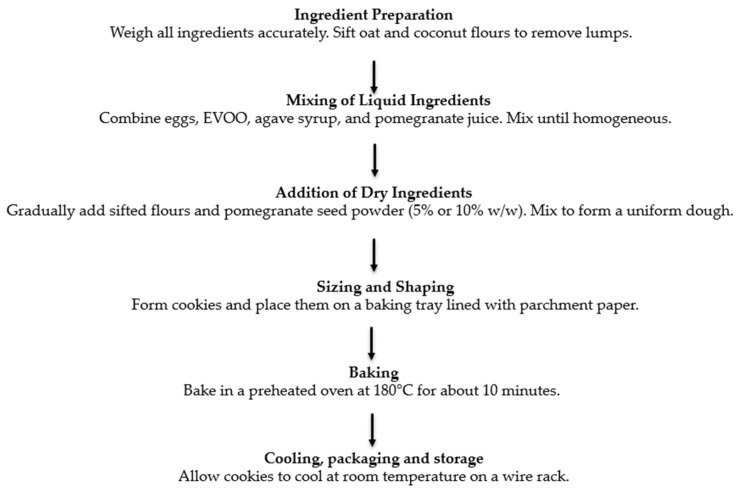
Flow chart for preparation of cookies.

Cookies were prepared using eggs, extra virgin olive oil (cv. Ottobratica, Belvedere Marittimo, Italy), whole oat flour (Conad, Bologna, Italy), coconut flour (Baule Volante, Bologna, Italy), agave syrup (Fior di Loto, cultivated in Mexico, packaged in Bologna, Italy), and pomegranate juice (Coop Italia S.C., Bologna, Italy), with the addition of pomegranate seeds powder (5% and 10% *w*/*w*). The preparation of gluten- and lactose-free cookies enriched with pomegranate by-products involved several sequential steps as reported in Figure 1.

All ingredients were weighed, and the flours (whole oat and coconut) were sieved to ensure uniform particle size and to remove any lumps. In a separate bowl, the liquid ingredients, eggs, extra virgin olive oil (*Olea europea* cv. Ottobratica), agave syrup, and pomegranate juice, were combined and mixed until a homogeneous emulsion was obtained using a blender (mod. LM 871 Perfect Mix + Moulinex, Milan, Italy) for 5 min.

Then, the dry ingredients were gradually incorporated into the liquid mixture, starting with the sifted flours, followed by the addition of pomegranate seeds powder at two inclusion levels (5% and 10% *w*/*w*). The resulting dough was mixed for approximately 3 min at room temperature (25 ± 2 °C) until reaching a uniform and workable consistency.

The dough was then portioned into small pieces (~16 g each, 5 × 5 cm) and shaped into discs using a spoon or mould. The discs were then placed on baking trays lined with parchment paper to prevent sticking. The cookies were baked in a preheated static oven (mod. Bertazzoni, La Germania, Ravenna, Italy) at 180 °C for approximately 10 min, until achieving a golden-brown surface. After baking, they were allowed to cool at room temperature (25 ± 2 °C) on a wire rack to prevent moisture condensation and maintain crispness.

**Figure 2:**
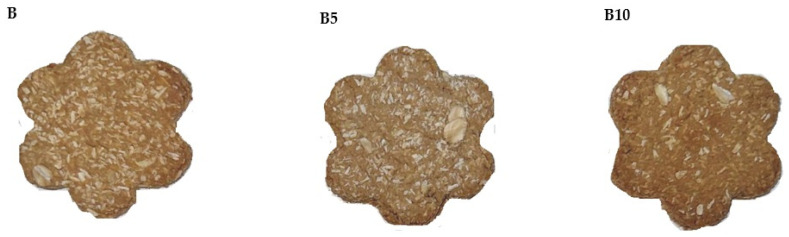
B: Control cookies; B5: Cookies enriched with 5% pomegranate seed flour; B10: Cookies enriched with 10% pomegranate seed flour.

About 40 cookies (each of 16 g) were obtained (Figure 2). Cookies were finely crushed for analysis, except for the textural and sensory analyses.

### 2.5. Determination of Energy Value

Cookie energy content was determined by adding the caloric contributions of proteins, carbohydrates, and fats for each ingredient, using label-reported nutritional values and accounting for the quantities used in the formulation. Conversion factors of 4 kcal/g for proteins and carbohydrates and 9 kcal/g for fats were used [27].

### 2.6. Determination of Macronutrient Content and Crude Fiber in PSF

Digestible carbohydrates were quantified using a modified analytical procedure adapted from Holm et al. [28]. Total fat content was determined by solvent extraction using the Majonnier method, as described by Croon and Guchs [29]. The Kjeldahl method was used to determines crude protein in flour from pomegranate seed by-products [27].

The crude fibre content of pomegranate powder (PSF) was determined using the Weende method [30], which involves sequential acid and alkaline digestion followed by incineration to quantify the insoluble residue. In the analysed samples (500 mg each), the crude fibre yield was 0.66% for sample A and 0.65% for sample B, indicating a consistent fibre recovery across replicates. These values align with previous studies reporting low crude fibre percentages in pomegranate by-products, though such residues are known to contain significant amounts of dietary fibre when analysed through more comprehensive methods such as Van Soest or enzymatic–gravimetric approaches [31,32]. The relatively low recovery obtained here can be attributed to the limitations of the Weende method, which underestimates soluble fibre fractions that are nutritionally relevant [33]. This analysis provides valuable insight into the properties of pomegranate powder, supporting its potential use as a functional ingredient in food formulations owing to the presence of bioactive fibre and associated phenolic constituents [34].

### 2.7. In Vitro Glycaemic Index Determination

The glycaemic index (GI) of cookies enriched with pomegranate by-products was determined using an in vitro digestion method adapted from Goñi et al. [35]. Briefly, finely ground biscuit samples (100 mg) were subjected to simulated gastric digestion with HCl–KCl buffer and pepsin, followed by intestinal digestion with α-amylase in sodium maleate buffer. Aliquots were collected at predetermined time points (0–180 min), thermally inactivated, cooled, and centrifuged. The supernatants were further hydrolysed with amyloglucosidase (E-AMGDF, Megazyme, Wicklow, Ireland), and the released glucose was quantified using a glucose oxidase/peroxidase (GOPOD, Megazyme, Wicklow, Ireland) assay. Absorbance was measured at 510 nm, and glucose values were converted to starch content. Starch hydrolysis was expressed as the percentage of total starch digested over time.

The hydrolysis index (HI) was calculated as the ratio between the area under the hydrolysis curve (0–180 min) of the tested sample and that of a reference food (white bread). The predicted glycaemic index (pGI) was obtained using the following equation:

pGI=0.549 × HI + 39.71

and subsequently converted to a glucose-based GI by multiplying pGI by 0.71.

### 2.8. Cookie Chemical–Physical Parameters

Cookie weight was recorded 2 h post-baking using a digital analytical balance (PCE-BSK 310, PCE Instruments, Capannori, Italy). Sample volume was determined following the AACC Method 10-91 [36].

Crumb moisture content was measured at 105 °C using a moisture analyser (MB, OHAUS, Zurich, Switzerland).

Soluble sugar content was quantified by digital refractometry (PR-201α, Tokyo, Japan; measurement range 0–60%) after homogenizing 5.0 g of sample with 50 g of distilled water for 5 min [37].

The colour differences were determined on the surface using a colorimeter (mod. CSM-4, PCE Instruments, Capannori (LU), Italy) as previously described [38].

### 2.9. Texture Analysis

The cookie’s texture was evaluated using two types of tests: the three-point bending test and the penetration test [39]. The structural tests used allowed the evaluation of important structural characteristics of the cookies, such as breaking strength and flexibility with the three-point bending test and hardness with the penetration test. Texture analysis was carried out using a TA-XT Plus texture analyser (Stable Micro Systems Ltd., Godalming, UK) equipped with a 50 kg load cell and a trigger force of 0.05 N, with data acquisition and analysis performed using Exponent software (v. 6.1.4.0). Measurements were conducted at room temperature (25 ± 5 °C) on ten cookies per formulation. A three-point bending test was performed using a three-point bend rig (A/3PB), applying a deformation distance of 5.0 mm with pre-test, test, and post-test speeds of 1.0, 3.0, and 10.0 mm/s, respectively. A penetration test was also conducted using a cylindrical P/2 probe (2 mm diameter) with a penetration depth of 3 mm and pre-test, test, and post-test speeds of 2.0, 1.0, and 1.0 mm/s, respectively.

### 2.10. Sensory Analysis

Sensory evaluation of pomegranate-enriched cookies was conducted at the sensory laboratory of the Department of Agriculture, Mediterranean University of Reggio Calabria using quantitative descriptive analysis. An eight-member trained panel of regular cookie consumers (5 men and 3 women, aged 23–56 years) was selected and trained according to ISO 8586:2012 guidelines [39]. The study complied with the Declaration of Helsinki, and all participants provided informed consent and reported no allergies or intolerances. Samples were presented in randomized order at room temperature in individual booths, with palate cleansing using natural mineral water between evaluations. Visual, olfactory, taste, and texture attributes were scored on an 11-point structured scale (0 = absence; 10 = extremely high), and results were expressed as mean values.

### 2.11. Pomegranate By-Product-Enriched Cookie Extraction Procedure

Ultrasound-assisted extraction was applied to the cookies using a hydroalcoholic solvent (ethanol/water, 9:1 *v*/*v*) at a 1:1 sample-to-solvent ratio as above previously described [25].

### 2.12. Total Phenolic Content (TPC) and Total Flavonoid Content (TFC)

Total phenolic content (TPC) was quantified using the Folin–Ciocalteu assay according to established procedures [25]. Results were expressed as mg gallic acid equivalents (GAE) per 100 g of dried weight for PSF and mg GAE/g cookie.

Total flavonoid content (TFC) was determined by a procedure previously described [25]. Data were reported as mg quercetin equivalents (QE) per 100 g of dried weight for PSF and mg QE/g cookie.

### 2.13. In Vitro Antioxidant Activity

#### 2.13.1. FRAP Assay

The ferric-reducing antioxidant power (FRAP) assay was carried out according to the procedure previously reported [25]. For the analysis, 2 mL of the FRAP solution were combined with 900 µL of distilled water and the extract (1.25 mg/mL). After a 30 min reaction at room temperature, absorbance was recorded at 595 nm using a UV–vis spectrophotometer (Jenway 6003, Milan, Italy). Butylated hydroxytoluene (BHT) was included as a reference antioxidant, and results were reported as µM Fe(II) equivalents per gram of sample.

#### 2.13.2. β-Carotene Bleaching Test

The ability of the extracts to inhibit lipid peroxidation was assessed using the β-carotene–linoleic acid model system [25]. A β-carotene emulsion was prepared by combining β-carotene, linoleic acid, and Tween 20. Extracts at concentrations between 2.5 and 100 µg/mL were added to the emulsion and incubated for 30, and 60 min. Absorbance was recorded at 470 nm using a UV–vis spectrophotometer against a blank. Results were expressed as IC_50_ values (µg/mL). Propyl gallate was employed as positive control.

#### 2.13.3. Evaluation of Radical Scavenging Potential by DPPH and ABTS Tests

The 2,2-diphenyl-1-picrylhydrazyl (DPPH) and 2,2′-azino-bis(3-ethylbenzothiazoline-6-sulfonic acid) diammonium salt (ABTS) assays were carried out as previously reported [25]. Ascorbic acid served as the positive control. Results were expressed as IC_50_ values (µg/mL).

### 2.14. Carbohydrate Hydrolyzing-Enzyme and Pancreatic Lipase Inhibitory Activity

The inhibitory activity of the samples against carbohydrate-hydrolysing enzymes was evaluated using adapted spectrophotometric assays [25]. α-amylase inhibition was determined by a modified DNS method using soluble starch as substrate, while α-glucosidase inhibition was assessed with p-nitrophenyl-α-D-glucopyranoside (pNPG). Reaction mixtures containing sample and enzyme were incubated at 37 °C, and absorbance was measured at 540 nm (α-amylase) or 405 nm (α-glucosidase) after reaction termination. Appropriate sample and reagent blanks were used for background correction, and inhibition percentages were calculated relative to controls. All analyses were carried out in triplicate, and the experiment was independently replicated three times. Acarbose was included as a reference inhibitor.

Pancreatic lipase inhibition was evaluated in a 96-well format using 4-nitrophenyl octanoate as substrate and porcine pancreatic lipase, following a modified protocol [40]. After incubation at 37 °C for 30 min, enzymatic activity was quantified by measuring *p*-nitrophenol release at 405 nm. Inhibition percentages were calculated after blank correction, with all assays performed in triplicate, and the experiment was independently replicated three times. Orlistat was used as the reference inhibitor.

### 2.15. Statistical Analysis

Data are presented as the mean ± standard deviation (SD) of triplicate measurements, and all experiments were independently repeated three times. IC_50_ values were calculated using GraphPad Prism (version 4.0). Statistical analyses were performed using one-way analysis of variance (ANOVA) followed by Tukey’s post hoc test with SPSS software (version 22.0). Differences were considered statistically significant at *p* < 0.05. Principal component analysis (PCA) and Pearson’s correlation analysis were also conducted.

## 3. Results and Discussion

### 3.1. Pomegranate Seeds Powder Chemical Profile

**Figure 3:**
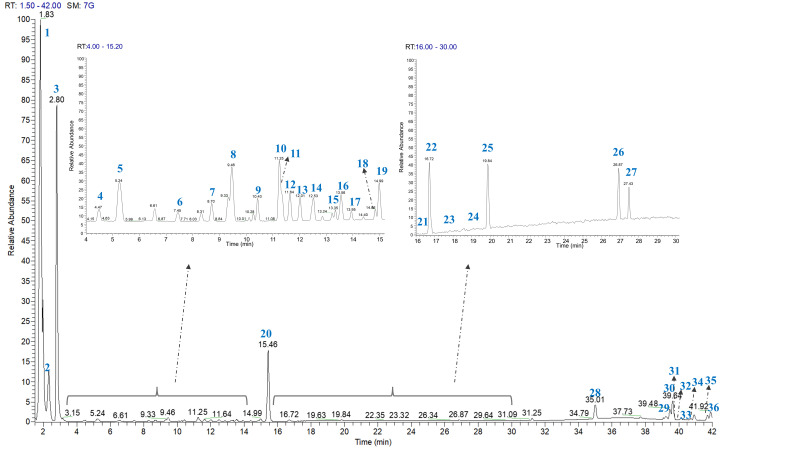
LC-ESI/HRMS profile of PSF extract in negative ion mode.

The chromatographic analysis of PSF revealed 36 major compounds, tentatively identified using accurate mass measurements, MS/MS fragmentation data, and literature comparison (Figure 3). The main analytical features of the identified compounds are summarized in Table 2.

All compounds were eluted within 42 min of chromatographic separation and mainly belonged to the subclasses of carbohydrates, flavonoids, phenolic acids, megastigmanes, and hydrolysable tannins, along with a lignan, a terpene, and polar lipids.

The chromatographic method enabled the detection of sucrose (**1**) and its derivative 2-(hydroxy-propyl)-sucrose (**4**), with *m*/*z* values of 341.1079 and 399.1503 Da, respectively. These compounds, previously identified in pomegranate, are known contributors to its natural sweetness [41,42].

Among the flavonoid derivatives, naringenin (**26**) was identified (Table 2), supported by its characteristic fragmentation patterns [41,43]. Additionally, glycosylated flavonoids such as dihydrokaempferol-*O*-hexoside (**17**) and kaempferol 3-*O*-rutinoside (**24**) were detected. Their MS/MS spectra showed base peaks produced by the neutral loss of sugar moieties: a dehydrated hexose (162 Da) for compound **17**, and combined losses of a dehydrated hexose and a dehydrated deoxyhexose (146 Da) for compound **24** [41,44,45]. All of these flavonoid derivatives have been previously reported in pomegranate extracts [41,44].

Organic acids such as citric acid (**3**) and its derivatives—methylcitric acid (**5**) and citric acid dimethyl ester (**8**)—were observed in the LC-ESI/HRMS/MS profile, as confirmed by MS/MS spectra and literature comparison. Citric acid (**3**) and its dimethyl ester (**8**) have been previously reported in pomegranate [41,43], whereas methylcitric acid (**5**) is reported here for the first time in a pomegranate extract.

Among the phenolic acids, gallic acid and hydroxycinnamic acid derivatives were also identified. Compounds **6** and **7** were characterized as vanillic acid-*O*-hexoside and syringic acid-*O*-hexoside, respectively, based on the neutral loss of a dehydrated hexose unit (162 Da) and the presence of diagnostic product ions corresponding to [(vanillic acid−H)]^−^ and [(syringic acid−H)]^−^. While vanillic acid-*O*-hexoside (**6**) has been previously reported in pomegranate [41], syringic acid-*O*-hexoside (**7**) is described here for the first time in this matrix. Moreover, compound **13** was identified as coumaric acid-*O*-hexoside [41].

Continuing the LC-ESI/HRMS/MS analysis, compounds **9** and **15** showed *m*/*z* values of 443.1918 and 431.1919 Da, respectively. In both cases, a major fragment ion was generated by the loss of 180 Da, corresponding to the loss of a hexose unit. Considering the observed fragmentation patterns together with literature data, these analytes were assigned to dihydrophaseic acid 3′-*O*-β-D-glucopyranoside and roseoside, megastigmane derivatives previously identified in pomegranate peel [46].

Furthermore, peak **10** at *m*/*z* 525.1612 Da was tentatively identified as demethyloleuropein, a terpene derivative previously found in pomegranate juice, based on its MS/MS fragmentation pattern and literature data [41].

Hydrolysable tannins represented one of the dominant polyphenolic classes in pomegranate extract, including gallotannins, ellagitannins, and ellagic acid derivatives. Compound **11** showed a [M−H]^−^ ion at *m*/*z* 785.0848 Da and exhibited a major fragment ion at *m*/*z* 300.9981 Da ([M−H−HHDP−glu−H_2_O]^−^) in the MS/MS spectrum. Comparison with previous studies [44] allowed its tentative identification as digalloyl-HHDP-glucoside (peduncalagin II). Compound **14** exhibited a deprotonated ion at *m*/*z* 633.0735 and yielded fragment ions at *m*/*z* 463.0521 and 301.9986, consistent with the sequential loss of gallic acid (170 Da) and glucose (162 Da) units. Additional fragments at *m*/*z* 275.0195 Da confirmed the presence of HHDP and gallic acid moieties, suggesting that compound **14** corresponded to one of the galloyl-HHDP-glucose isomers [44].

Compound **12** exhibited a [M−H]^−^ ion at *m*/*z* 799.0648 and characteristic fragment ions at *m*/*z* 300.9985 (ellagic acid unit) and 273.0041 (subsequent CO loss), supporting its assignment as granatin A. Compound **19** showed a [M−H]^−^ ion at *m*/*z* 951.0783 with analogous fragment ions (*m*/*z* 300.9987 and 273.0045) and was therefore identified as granatin B [44]. Among ellagic acid derivatives, compounds **16** and **21** displayed [M−H]^−^ ions at *m*/*z* 463 and 433, respectively, and MS/MS fragments at *m*/*z* 301, indicative of hexose and pentose losses, leading to their tentative identification as ellagic acid-*O*-hexoside (463.0518) and ellagic acid-*O*-pentoside (433.0410). Ellagic acid (**23**) was further confirmed by its diagnostic fragment ions at *m*/*z* 284.0511 and 229.0136 [44]. These hydrolysable tannins have been previously reported in pomegranate matrices [41,43,44]

Additional LC–ESI–HRMS analyses indicated the presence of a lignan-type compound. Compound **22** was characterized by the molecular formula C_24_H_28_O_12_, and its MS/MS spectrum displayed a product ion at *m*/*z* 327.0869 ([M−H−180]^−^), consistent with the cleavage of a hexose moiety.

This information, supported by the literature, allowed its identification as pomegralignan, previously isolated from pomegranate arils [47]. In addition, peak **18** (C_19_H_28_O_10_) was tentatively identified as icariside D1, a phenethyl rutinoside previously isolated from pomegranate seeds [18].

The dominant signal in the LC–ESI–HRMS/MS chromatogram was attributed to compound **20**, showing a deprotonated ion at *m*/*z* 475.1806. Based on its molecular formula (C_21_H_32_O_12_), MS/MS fragmentation pattern, and comparison with published data, this compound was tentatively identified as kanokoside A, an oligopeptide previously reported in pomegranate matrices [44]. Compound **28** exhibited an *m*/*z* value consistent with the molecular formula C_17_H_26_O_4_; its MS/MS spectrum, in agreement with literature reports, supported its assignment as 6-gingerol, a compound earlier detected in pomegranate extracts [43].

Finally, polar lipids were identified in pomegranate for the first time. These included stearic acid (**25**), oxylipins (**27** and **31**), a glycolipid (**32**), and lysophospholipids (**29**, **30**, **33**–**36**). Characterization of these compounds was based on detailed MS/MS spectral analysis, which revealed diagnostic product ions specific to each lipid class. Compounds **27** and **31** were identified as oxylipin derivatives—oxygenated lipid molecules formed via singlet oxygen- or dioxygen-mediated oxidation pathways. Analysis of compounds **29**, **33**, and **35** indicated that they belonged to the lysophosphatidylethanolamine (L-PE) class, based on their molecular formulas (containing NO_7_P) and diagnostic ions at *m*/*z* 214 and 196 Da, corresponding to the lipid head group and glycerophosphatidyl unit (intact or mono-dehydrated) [48]. Compounds **30**, **34**, and **36** were tentatively classified as lysophosphatidylcholine (LPC) derivatives based on the presence of a characteristic fragment ion at *m*/*z* 224, attributed to a mono-dehydrated glycerol–phosphatidylethanol(dimethyl)amine moiety [49]. In negative ionization mode, their MS/MS spectra were further characterized by a predominant neutral loss of 60 Da (C_2_H_4_O_2_) from the precursor ion.

Furthermore, LC-ESI/HRMS/MS analysis allowed compound **32** to be classified within the glycolipid family (Table 3, Figure 3). Specifically, compound **32** was identified as monogalactosyl-monoacylglycerol (MGMG), based on the presence of a characteristic product ion at *m*/*z* 253.0919 Da, corresponding to the monogalactosylglycerol moiety [48]. In all of these polar lipids, the fatty acid chain composition could be inferred from the presence of a dominant RCOO^−^ product ion [48].

### 3.2. Nutritional Characteristics of Pomegranate Seed By-Products (PSF) and PSF-Enriched Cookies

Pomegranate seed flour (PSF) can be considered as a nutrient-dense matrix, showing a moderate protein content (4.67%), a relevant lipid fraction (11.49%), and appreciable carbohydrate levels (18.74%). These compositional features are fully consistent with previously reported data, confirming the intrinsic variability of PSF while supporting the reliability of the present results (Table S1, Supplementary Materials).

Table 3 showed the energy value of control cookies (B), and functionalized cookies with 5 and 10% *w*/*w* of pomegranate seed flour (B5 and B10, respectively).

Cookies enriched with pomegranate seed flour (PSF) showed a slight increase in caloric value (377.30 and 376.20 kcal for B5 and B10, respectively) when compared with the control (B, 376.20 kcal). However, these differences were not statistically significant, nor were the variations in carbohydrates, fats, proteins, or fibre content. Considering that breakfast typically contributes a portion of daily energy intake, the developed product, providing approximately 150–200 kcal per serving, may represent a fibre-enriched option suitable for inclusion within a balanced breakfast, when consumed as part of a varied diet and in combination with other nutrient-dense foods, rather than as a standalone meal [50].

From a nutritional standpoint, the enrichment with PSF produced only minor variations in macronutrient composition, with a slight increase in fibre content when compared with the control cookies. These results are consistent with previous evidence showing that fruit-derived by-products can enhance the fibre and phytochemical profile of bakery products with minimal impact on caloric value [8,51]. The limited changes observed here, likely related to the moderate inclusion levels (5–10%), further confirm that PSF can be incorporated into gluten- and lactose-free formulations without altering their overall nutritional balance.

**Figure 4:**
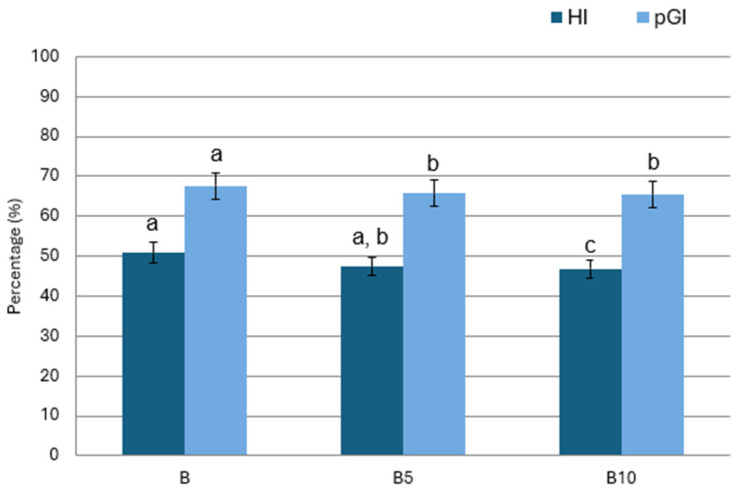
Results of predicted glycaemic index (pGI) and hydrolysis index (HI) of cookies. B: Control cookies; B5: Cookies enriched with 5% pomegranate seed flour; B10: Cookies enriched with 10% pomegranate seed flour. Data are shown as mean of triplicate and the experiment was independently replicated three times ± SD and analysed by one-way ANOVA followed by Tukey’s multiple comparison test. Different lowercase letters indicate a significant difference (p < 0.05) among samples for pGI and HI, respectively.

In the present study, the hydrolysis index (HI) and predicted glycaemic index (pGI) of PSF-enriched cookies were evaluated, and the results are shown in Figure 4. The control cookies exhibited an HI value of 50.08, while lower values were observed for the PSF-enriched samples, namely 47.4 and 46.8 for the B5 and B10 formulations, respectively. A similar trend was observed for pGI, which decreased from 67.6 in the control sample to 65.4 in the B10 cookies. Generally, all formulations can be classified as low-GI foods (GI < 55), showing lower values than those typically reported for conventional cookies, which exhibit high GI levels (70–80) [52]. The observed reduction in pGI may be partially attributed to the higher dietary fibre content of the PSF-enriched formulations.

It should be noted, however, that the glycaemic index values reported in this study were obtained using an in vitro digestion model. Although such models are commonly employed for preliminary screening and comparative assessment, they present several limitations. In vitro pGI determinations are not fully standardized and are unable to reproduce the complexity of human digestion, including mastication, gastric emptying rate, intestinal motility, hormonal regulation, and inter-individual metabolic variability. Consequently, the correlation between in vitro-predicted GI and in vivo GI values may be limited, particularly for complex food matrices such as fibre-enriched baked products [53]. These limitations may lead to under- or overestimation of the actual postprandial glycaemic response and, in some cases, to misclassification of foods as low-GI. Therefore, although the lower pGI values observed for PSF-enriched cookies suggest a potentially beneficial glycaemic impact, in vivo human intervention studies are required to confirm these findings and to support accurate GI labelling.

The reduction in carbohydrate hydrolysis observed in the present study is consistent with previous literature. Dartois et al. [54] have reported that soluble dietary fibre (SDF) reduces the extent of carbohydrate hydrolysis by α-amylase by increasing the viscosity of the digested medium. Similarly, García et al. [55] showed that the replacement of 2.5–5% of wheat flour with pomegranate peel powder significantly increased dietary fibre and polyphenol contents, resulting in a reduced GI while maintaining good sensory acceptability. Moreover, the reduction in pGI, observed in our samples, agrees with other studies attributing lower glycaemic index values to increased matrix complexity and to the high content of insoluble dietary fibre, which is known to slow starch hydrolysis.

Recent studies have also highlighted the potential of pomegranate-derived ingredients to improve the nutritional quality of baked products. Harish et al. [56] demonstrated that the incorporation of PSF into cookies increased crude fibre content by up to 17.10%, with minimal effects on caloric value and major macronutrients. Similarly, Kavitha et al. [57] have reported that the addition of pomegranate peel and grape must powder markedly increased dietary fibre content while only marginally affecting total energy value. Comparable results were observed in cookies enriched with pomegranate seed flour, which also showed negligible changes in total energy content. In addition, Askar [58] reported that the incorporation of pomegranate peel powder improved both fibre and mineral contents without significantly influencing the caloric value of cookies.

Overall, these findings suggest that the incorporation of pomegranate-based ingredients into cookie formulations represents an effective strategy to enhance nutritional quality, particularly dietary fibre content, while maintaining a balanced energy profile. Consequently, PSF-enriched cookies may be considered promising functional baked products with potential health benefits.

### 3.3. Physical Characteristics and Sensory Profile of Cookies

Mechanical testing demonstrated that the incorporation of pomegranate flour markedly influenced cookie structure. Breaking force indicates the effort needed to snap the cookie and is therefore linked to how crisp and resistant it feels when bitten. Hardness describes how firm the cookie feels during the first bite, because of how strongly it resists pressure and deformation. Flexibility refers to the cookie’s ability to bend slightly before breaking and is perceived as a lower tendency to crumble and a more yielding, less brittle texture during chewing. As regards the three-point bending test, the enrichment of the sample B5 did not show significant variations either in the breaking force or in the flexibility when compared with the control sample (B), with values of 38.27 N and 1.68 mm, and 35.37 N and 1.06 mm, respectively. At the highest enrichment level (10%, B10), a significant reduction in breaking force (21.72 N; *p* < 0.01) was observed together with an increase in flexibility (2.09 mm), suggesting a more deformable and less stable structure (Table 4).

Textural changes can be attribute to the interaction effects related to PSF incorporation.

Among these variables, PSF contains a high content of fibre that may interrupt the gluten-free structure, reducing resistance and cohesion to mechanical stress. PDF’s fibre can increase water-binding and reduce the homogeneous structure of the gluten-free product, reducing the hardness and increasing the flexibility of the biscuits. Furthermore, PSF contains a lipid fraction rich in unsaturated fatty acids [14], which can contribute to the reduction of hardness and act as a plasticizer [59]

Collectively, these outcomes highlight the significant role of pomegranate waste in shaping product structure, probably driven by its fibre-rich composition and related rheological effects [60].

**Figure 5:**
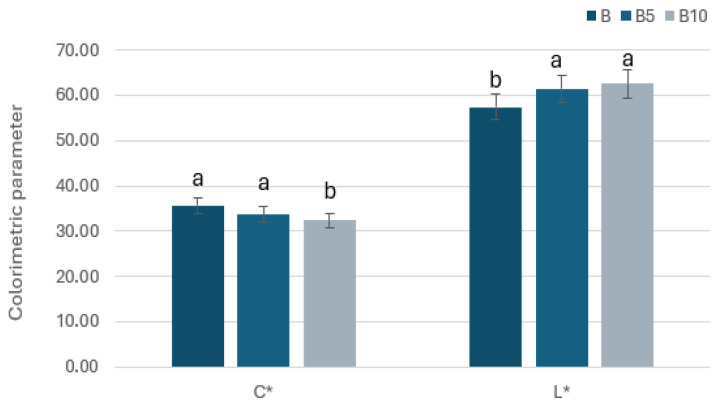
Chroma (C*) and luminosity (L*) in different cookie formulations. B: Control cookies; B5: Cookies enriched with 5% pomegranate seed flour; B10: Cookies enriched with 10% pomegranate seed flour. Differences between samples were evaluated by one-way ANOVA followed by Tukey’s test. Results followed by different letters are significantly different at p < 0.01.

The addition of two different percentages of pomegranate seed flour to the cookies resulted in a reduction of the chroma (C*) value, while no significant differences were observed in the lightness (L*) parameter between the control sample and the cookies enriched with 5% and 10% flour (Figure 5). Our findings on L* disagree with those reported by Ayoubi et al. [61] that indicated that an increase in pomegranate seed flour addition reduced the luminosity.

ΔE values of 147.17 and 490.5 for B5 and B10, respectively, were observed, demonstrating that the sample with the highest level of enrichment exhibited the most significant colour change relative to the control.

The colour of baked products is strongly influenced by the inherent pigmentation of the raw materials, which can result in a darker brown hue in the final product. Beyond the raw ingredients, product formulation plays a pivotal role in the development of browning during baking. Several parameters, including sugar composition, water activity, and temperature during processing, play a key role in the formation of coloured compounds.

As reported by Purlis et al. [62] and Sangnark and Noomhorm [63], the addition of dietary fibre tends to darken the colour of baked goods, whereas the incorporation of proteins can accelerate the Maillard reaction. This, in turn, promotes the formation of melanoidins, yielding a more intensely browned crust. These findings underscore the interplay between ingredient composition and processing conditions in determining the final appearance and quality of baked products. In conclusion, the addition of PSF at low levels (5–10%) can subtly modify cookie colour without compromising overall brightness, likely due to the presence of phenolic compounds that influence visual perception.

### 3.4. Sensory Analysis Results

**Figure 6:**
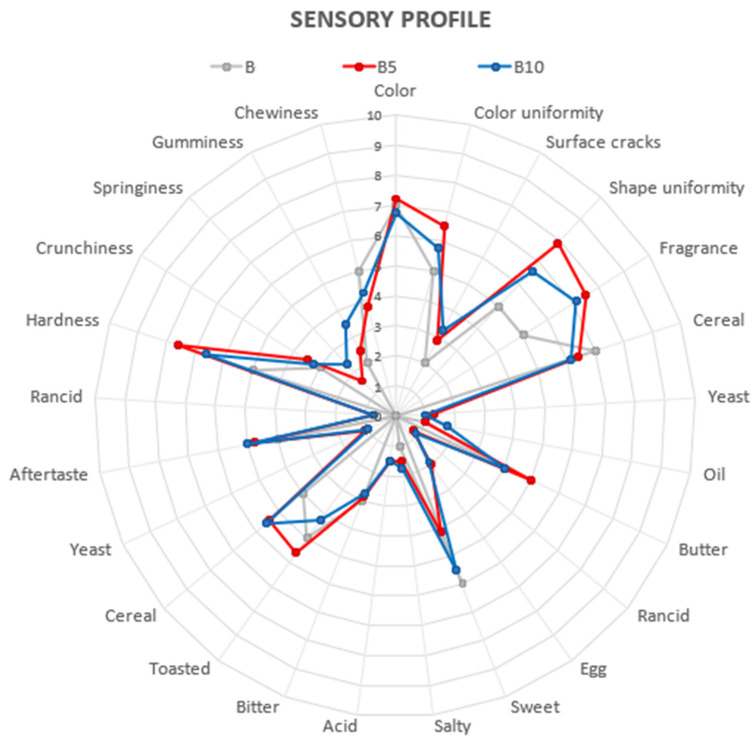
Sensory profile spider plot: B: Control cookies; B5: Cookies enriched with 5% pomegranate seed flour; B10: Cookies enriched with 10% pomegranate seed flour.

Panel results were processed and rendered in graphical form using spider plots. Sensory data showed a sample sensory profile (Figure 6).

In the sensory profile, the main descriptors identified by panellists were colour, shape, fragrance and cereal aroma, sweet, toasted and cereal taste, aftertaste and textural hardness.

Enriched cookies (B5 and B10) showed a significantly higher sensory profile behaviour than the control sample, with an increase in almost all descriptors, except for cereal aroma, sweet taste, and textural chewiness.

Texture descriptors accounted for the main sensory differences between control and fortified samples, characterized by an increase in perceived hardness and a decrease in chewiness. Among the enriched cookies, B5 displayed significantly higher ratings for visual attributes (colour, colour uniformity, and shape uniformity) and for toasted flavour compared with B10.

Most structural texture sensory attributes did not differ significantly among samples, except for springiness and gumminess, which were higher in B10. Consistent with these findings, Bhise et al. [64] reported that cookies enriched with pomegranate seed flour (5–20%) showed optimal baking quality and sensory performance at 15% inclusion. Similarly, the incorporation of pomegranate seed powder into cupcake formulations (2.5–10%) demonstrated that additions up to 5% did not negatively affect sensory properties during refrigerated storage [59 Ayoubi].

The behaviour of the food matrix observed in this study is consistent with what has been reported for baked goods enriched with PSF or pomegranate peel powder (PPP).

Gül & Şen [65] showed that the inclusion of 5% PSF in bread leads to rheological and structural changes, with increased hardness, volume reduction and chromatic alterations, while maintaining an improved nutritional profile. However, it is important to notice that the instrumental parameters of texture do not directly correspond to the sensory perception of texture. Mechanical characteristics are complementary to sensory analysis, the latter strongly influenced by various human perception factors [66].

### 3.5. Bioactive Compounds in Pomegranate Seed By-Product (PSF) and PSF-Enriched Cookies

The total phenolic content (TPC) and total flavonoid content (TFC) of pomegranate seed by-products were 0.09 mg quercetin equivalents (QE) per 100 g of dry weight and 0.05 mg gallic acid equivalents (GAE) per 100 g of dry weight, respectively (Table S1).

**Figure 7:**
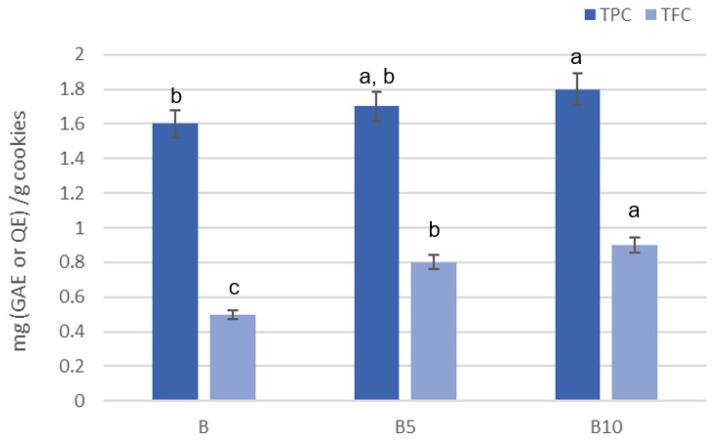
Total phenol content (TPC) and total flavonoid content (TFC) in B: Control cookies; B5: Cookies enriched with 5% pomegranate seed flour; B10: Cookies enriched with 10% pomegranate seed flour. Differences between samples were evaluated by one-way ANOVA followed by Tukey’s test. Results followed by different letters are significantly different (p < 0.01).

TPC values were similar across formulations, ranging from about 1.6 to 1.8 mg GAE/g cookie, with no statistically significant differences (*p* > 0.05) (Figure 7). The TFC was consistently lower than TPC in all samples (*p* < 0.05) and showed no consistent relationship with increasing pomegranate powder levels (Figure 7).

Overall, TPC values remained comparable among the different cookie’s formulations, while TFC values did not show a clear relationship with increasing levels of PSF enrichment. These findings suggest that PSF contributed to the bioactive compound content of cookies, although the baking process may have limited the overall retention of phenolics and flavonoids.

Phenolic compounds are known to exhibit limited thermal stability at temperatures around 180 °C, which are typical of baking processes, leading to substantial losses because of oxidation, cleavage of phenolic bonds, and interactions with other components of the food matrix [67]. Among these compounds, pomegranate ellagitannins, particularly punicalin and punicalagin, are highly sensitive to intensive thermal treatments [68,69]. Previous studies have demonstrated that increasing temperature and heating duration promote hydrolysis and degradation of these molecules, with the concomitant formation of ellagic acid and a marked reduction in overall antioxidant capacity. Although punicalagin, due to its more complex and polymeric structure, generally shows slightly higher thermal stability than punicalin, both compounds can undergo degradation exceeding 50% under severe heating conditions, with punicalin degrading more rapidly [70]. While factors such as oxygen availability, moisture content, and the food matrix may partially modulate degradation rates, at high temperatures the effect of heat remains predominant, rendering such processing conditions unfavourable for the preservation of pomegranate ellagitannins.

In line with this evidence, the incorporation of PSF into the cookie formulations resulted in only a slight increase in TPC and TFC when compared with the control, with no statistically significant differences (*p* > 0.05). This suggests that PSF represents a source of bioactive compounds, the baking process substantially limited the retention of phenolics and flavonoids within the final product. Similar trends have been reported for other phenolic-rich ingredients subjected to high-temperature baking, where thermal degradation and matrix interactions outweigh the initial enrichment effect [71]. Therefore, the modest enhancement observed in the enriched cookies can reasonably be attributed to the extensive thermal stress imposed during baking.

From a kinetic perspective, the degradation of pomegranate ellagitannins is commonly described by first-order reaction models. Studies performed on pomegranate juices and model systems have shown that punicalagin degradation follows first-order kinetics, with rate constants increasing exponentially with temperature. For instance, Fischer et al. [70] reported rate constants in the range of 0.003–0.006 min^−1^ at 80–90 °C, corresponding to half-lives between 110 and 230 min, indicating moderate stability at lower temperatures but a pronounced acceleration of degradation as temperature increases. Although experimental kinetic data at 180 °C are scarce, Arrhenius-based extrapolations suggest a drastic reduction in half-life to only a few minutes, consistent with losses exceeding 50–70% even during relatively short baking treatments. Punicalin, characterized by a less complex molecular structure, exhibits higher degradation rate constants and consequently lower thermal stability when compared with punicalagin. Reported activation energy values for pomegranate ellagitannins (approximately 60–90 kJ·mol^−1^) further confirm their high sensitivity to heat [72,73].

Overall, these findings indicate that severe thermal processing conditions, such as baking at 180 °C, are largely incompatible with the effective preservation of punicalin and punicalagin in baked products. While the food matrix and limited oxygen diffusion in dough systems may partially mitigate degradation, these protective effects appear insufficient to counterbalance the dominant impact of high temperature [74].

From a technological perspective, this highlights the need for alternative formulation or processing strategies such as lower baking temperatures, shorter baking times, or post-baking fortification to enhance the retention of pomegranate-derived phenolic compounds in bakery products.

Recent studies have reported the formulation of muffins enriched with pomegranate peel (PP), an agro-industrial by-product, showing a marked increase in total phenolic content, from 0.443 mg GAE/100 g in control samples to 48.53 mg GAE/100 g in muffins containing 8% PP [75]. These findings suggest that such bakery products exhibit an antioxidant potential comparable to that observed in the formulations investigated in the present study [76]. Likewise, Nakow et al. [77] observed a significant enhancement in total phenolic content in cookies fortified with 6% grape pomace relative to the control formulation. In line with these findings, the addition of PSF also resulted in darker coloration, increased friability, and greater structural density in our cookies.

Previous studies attribute these effects to the higher content of insoluble fibre and polyphenols, which influence water absorption and structure formation during baking [55,78]. Despite these changes, overall acceptability remained positive even at the 10% level; this is a particularly significant result, considering that the literature reports a reduction in acceptability already exceeding 5% in baked goods with pomegranate by-products (peel powder) [55].

### 3.6. Antioxidant Activity of PSF By-Product and PSF-Enriched Cookies

A combination of complementary in vitro assays (DPPH, ABTS, FRAP, and β-carotene bleaching) was applied to assess the antioxidant capacity of PSF and enriched cookies. The radical scavenging activity of PSF against DPPH and ABTS radicals resulted in a moderate inhibition, with percentages of 42.58% and 58.99%, respectively, at concentration of 500 μg/mL (Table S1). A more pronounced protective effect against lipid peroxidation was observed with percentages of inhibition of 61.31 and 63.88% at 100 μg/mL after 30 and 60 min incubation, respectively, while the resulting FRAP value was 8.30 μM Fe (II)/g (Table S1).

The corresponding results for the control (B) and the enriched cookie formulations (B5 and B10) are presented in Table 5. The incorporation of pomegranate by-product improved the antioxidant potential of the cookies in all tests performed.

The FRAP assay demonstrated significantly higher reducing activity in enriched samples, reaching 67.82 μM Fe (II)/g in B10 compared with 55.83 μM Fe (II)/g in the control (*p* < 0.05). Noteworthy, the cookie’s FRAP results are of the same order of magnitude as those of the positive control BHT.

Similarly, in the β-carotene bleaching test, the enriched cookies exhibited lower IC_50_ values when compared with the control (16.03 and 12.72 μg/mL vs. 41.69 and 31.38 μg/mL for B10 and B at 30 and 60 min of incubation, respectively), confirming their improved ability to inhibit lipid peroxidation. However, the observed inhibition of lipid peroxidation was approximately two orders of magnitude lower than that achieved by the reference antioxidant propyl gallate used as positive control.

In the DPPH radical scavenging assay, the IC_50_ values decreased progressively from 31.38 μg/mL in the control to 12.72 μg/mL in B10, indicating a concentration-dependent enhancement of free radical quenching activity. A similar trend was observed in the ABTS assay, where the percentage of inhibition at 1 mg/mL increased from 20.56% in the control to 32.04% in the B10 formulation, while the IC_50_ value decreased from 549.01 μg/mL to 406.53 μg/mL. In both cases, the activity was significantly lower than that of the ascorbic acid used as positive control.

Correlation analysis revealed a positive association between phenolic and flavonoid contents and antioxidant activity across the cookie samples, with *r* values of 0.97, 1.0, 0.98, 0.82, 0.99 and 1.0, 0.99, 1.0, 0.77, 0.86 for FRAP, the β-carotene bleaching tests at 30 and 60 min of incubation, DPPH and ABTS, respectively.

Overall, the enrichment with PSF, particularly at 10%, enhanced the antioxidant properties of the cookies. These findings may be explained by the presence of polyphenols and flavonoids naturally occurring in pomegranate seeds, which function as effective hydrogen- or electron-donating agents, thereby interrupting free radical chain reactions. Nevertheless, the antioxidant capacity of the cookies was lower than that of the pure reference antioxidants (ascorbic acid, BHT, and propyl gallate), which is expected given the complexity of food matrices. Previous studies have documented the antioxidant potential of pomegranate-derived products. Jing et al. [78] reported marked differences in phytochemical composition and antioxidant activity among four Chinese pomegranate cultivars, with activity ranked as Suanshiliu > Tianhongdan > Sanbaitian ≈ Jingpitian. Similarly, cookies fortified with 3–5% pomegranate peel powder exhibited increased phenolic content and antioxidant capacity without adversely affecting their rheological properties [79].

Some pomegranate constituents, mainly ellagitannins, flavonoids, granatins, lignans and polar lipids have shown an interesting antioxidant activity [80].

Tannins, such as punicalagins and ellagic acid, are metabolized by gut bacteria into urolithins, which readily enter the systemic circulation and exhibit a significant antioxidant activity correlated with the number of hydroxyl groups as well as lipophilicity of the molecules [81]. Specifically, punicalagin exhibited IC_50_ values of 109.53 and 151.50 μg/mL in DPPH and the ABTS test, respectively [82]. A marked DPPH free radical scavenging activity was observed for pedunculagin, which exhibited a low IC_50_ value of 2.41 µM [83] and for both granatin A and B (IC_50_ values of 3.18 and 3.71 µM, respectively) [84]. In contrast, the lignan roseoside displayed a markedly weaker scavenging effect, with an IC_50_ of 41.3 µM [85]. This substantial difference in activity highlights the superior radical-scavenging efficiency of ellagitannins when compared with roseoside, likely reflecting structural features that enhance its ability to donate electrons or hydrogen atoms to neutralize free radicals.

Kaempferol and its derivatives, identified in pomegranate seed extract, are flavonols that are able to reduce the lipid oxidation in the human body, to prevent the organs and cell structure from deterioration and to protect their functional integrity [86].

Among the PSF-identified compounds, a promising radical-scavenging effect was also found for 6-gingerol against superoxide, DPPH, ABTS, and hydroxyl radicals [87,88].

Regarding polar lipids, Lucci et al. [19] demonstrated that the ethanolic extract of *P. granatum* cv. Dente di Cavallo seeds exhibited a threefold higher protective effect against lipid peroxidation compared with the positive control. This extract contained lipid compounds, such as neutral lipids (punicic acid 72.8%), glycolipids and phospholipids rich in essential fatty acids. Punicic acid is characterised by hydroxyl radical scavenging activity, metal chelation and reducing properties. Moreover, the presence of phospholipid rich in polyunsaturated fatty acids could not only contribute to the antioxidant activity but also act synergistically with phenolic antioxidants [89].

### 3.7. In Vitro Carbohydrate Hydrolysing Enzymes and Lipase Inhibitory Effects by Pomegranate By-Product-Enriched Cookies

Pomegranate by-products (PSF) and enriched cookies were investigated for their ability to inhibit carbohydrate hydrolysing enzymes (α-amylase and α-glucosidase) and pancreatic lipase (Table S1 and Table 6).

PSF exhibited a promising enzymatic inhibitory activity, showing the highest inhibition against α-amylase (87.38%), followed by α-glucosidase (57.25%). A moderate inhibitory effect was observed against pancreatic lipase (32.33%) at 1000 μg/mL (Table S1).

The incorporation of PSF markedly influenced the inhibition of these key enzymes associated with carbohydrate and lipid metabolism. The incorporation of PSF may contribute to the modulation of inhibitory effects on digestive enzymes associated with carbohydrate and lipid metabolism.

Analysing the α-glucosidase inhibitory activity, IC_50_ values significantly decreased from 1.91 mg/mL for the control cookie (B) to 0.36 mg/mL for B10 (*p* < 0.05), indicating that the enriched samples were more effective.

A similar trend was observed for α-amylase, where B5 and B10 exhibited substantially lower IC_50_ values (0.37 and 0.25 mg/mL, respectively) compared with the control (1.18 mg/mL, *p* < 0.05), confirming a concentration-dependent enhancement of inhibitory potential. In each case, B10 exhibited a ~10- and 5-time lower activity than the positive control acarbose against α-amylase and α-glucosidase, respectively.

Regarding lipase inhibition, B10 exhibited the strongest activity (IC_50_ value of 0.79 µg/mL), which was higher than that of the cookie control (0.98 µg/mL) and B5 (0.97 µg/mL) (*p* < 0.01). However, this activity is significantly lower than the reference compound orlistat.

This effect suggests that the bioactive compounds from PSF, particularly polyphenols and unsaturated fatty acids, may interact with enzyme active sites, reducing substrate accessibility.

Overall, the enrichment with pomegranate by-products, particularly at the 10% level, improved the cookies’ potential to modulate key metabolic enzymes involved in glucose and lipid absorption. These findings suggest that the fortified cookies may represent a promising candidate for functional food development, with potential relevance for the dietary management of hyperglycaemia and obesity. Previously, Thulsidhar et al. [90] developed cookies enriched with pomegranate seed powder (PSP) and defatted soybean flour (DSF) and evaluated their effects on serum glycaemic activity in diabetic and non-diabetic rats administered 5 g or 10 g/rat/day for 21 days. Their findings showed that the G6 group (standard diet + 10 g cookies/rat/day) exhibited the most pronounced improvements, with reductions of 32.43% in serum glucose and 36.99% in cholesterol compared with baseline. The combined addition of PSP and DSF to cookies led to improvements in glycaemic and lipid profiles, along with increased haemoglobin levels, in both diabetic and non-diabetic rats, without adversely affecting physiological parameters at any dose. The positive effects of PSF on metabolic syndrome have been confirmed by several authors [91,92,93]. Seyed Hashemi et al. [94] further observed marked reductions in circulating cholesterol concentrations in individuals with type 2 diabetes mellitus following twice-daily intake of 5 g pomegranate seeds powder. According to Elbandy and Ashoush [95], the phytochemical profile of pomegranate seeds plays a key role in modulating lipid metabolism, thereby contributing to their lipid-lowering and hypocholesterolaemia effects.

Several compounds identified in PSF have been reported as effective inhibitors of key digestive enzymes involved in carbohydrate and lipid metabolism, including α-amylase, α-glucosidase, and pancreatic lipase. Among these, punicalagin and punicalin, the major ellagitannins in PSF, display notable inhibitory activity due to their high phenolic content and abundance of hydroxyl groups. These structural features facilitate interactions with digestive enzymes through hydrogen bonding and hydrophobic interactions, leading to competitive or mixed-type inhibition [96]. Punicalagin, punicalin, and ellagic acid have been shown to inhibit α-glucosidase activity, exhibiting IC_50_ values of 140.2, 191.4, and 380.9 μM, respectively. Kinetic analyses suggested a mixed-type inhibition mechanism, indicating interactions with both the free enzyme and the enzyme–substrate complex. The inhibitory activity was confirmed under simulated gastrointestinal conditions using a starch-rich food matrix, supporting the physiological relevance of these compounds. Pre-incubation with α-glucosidase enhanced inhibition, consistent with direct enzyme binding. Although digestion reduced punicalagin and punicalin levels, the pomegranate extract maintained strong inhibitory activity, likely due to bioaccessible metabolites and synergistic phenolic interactions. Among ellagitannins identified in PSF, pedunculagin deserves particular attention due to its potent α-glucosidase inhibitory potential (IC_50_ value of 0.46 μM) as well as granatin A and B (IC_50_ values of 0.67 and 0.38 μM, respectively) [84].

Overall, these findings suggest that pomegranate ellagitannins may contribute to the modulation of postprandial glycaemia, lipid digestion, and the lipid-lowering and anti-obesity effects associated with pomegranate-based foods [97].

### 3.8. Principal Component Analysis

The relationships among cookie formulations and the measured chemical, functional, textural, and sensory parameters were evaluated by principal component analysis (PCA). The first two principal components explained most of the data variability, with PC1 accounting for 56.75% and PC2 for 43.25% of the total variance.

The score plot (Figure S1) showed a clear discrimination between the control sample (B) and the cookies enriched with pomegranate seed flour (B5 and B10), confirming that enrichment significantly affected the overall product profile. The enriched samples were progressively distributed along the principal components according to the increasing level of pomegranate seed flour, indicating a dose-dependent effect, with B10 showing the greatest separation and B5 assuming an intermediate position between B and B10.

**Figure 8:**
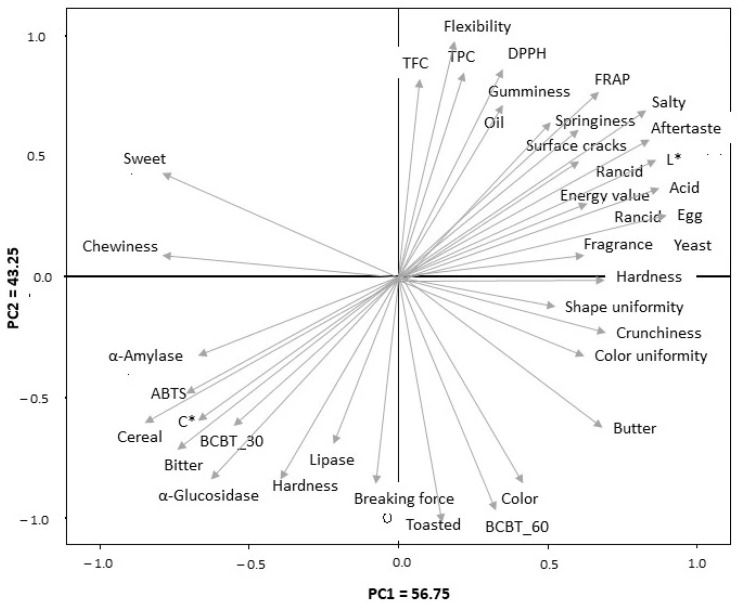
Loading plot of the principal component analysis (PCA) of chemical, functional, textural and sensory variables. TPC: Total phenol content; TFC: Total flavonoid content; BCBT_30: β-Carotene bleaching test results at 30 min incubation; BCBT_60: β-Carotene bleaching test results at 60 min incubation.

The loading plot (Figure 8) revealed that PC1 was mainly associated with structural, compositional, and sensory attributes. Positive loadings were observed for hardness, crunchiness, shape and colour uniformity, acidic and rancid notes, aftertaste, and energy value, whereas negative loadings were related to enzymatic inhibitory activities (α-amylase, α-glucosidase, and lipase), ABTS antioxidant capacity, and sensory attributes such as bitterness and cereal notes.

In contrast, PC2 was predominantly influenced by functional and antioxidant parameters, showing high positive loadings for total phenolic content, total flavonoid content, and antioxidant capacity evaluated by DPPH and FRAP assays, together with oil flexibility and gumminess, which were strongly associated with the B10 formulation. Negative PC2 loadings were mainly related to mechanical texture parameters and colour- and toasting-related attributes.

Overall, PCA highlighted that control cookies were mainly characterized by conventional sensory properties, whereas the incorporation of PSF enhanced the functional and antioxidant characteristics of the products. The 10% formulation showed the strongest association with bioactive compounds and antioxidant capacity, while the 5% formulation represented a suitable compromise between functional improvement and acceptable sensory and textural quality.

### 3.9. Correlation Matrix

**Figure 9:**
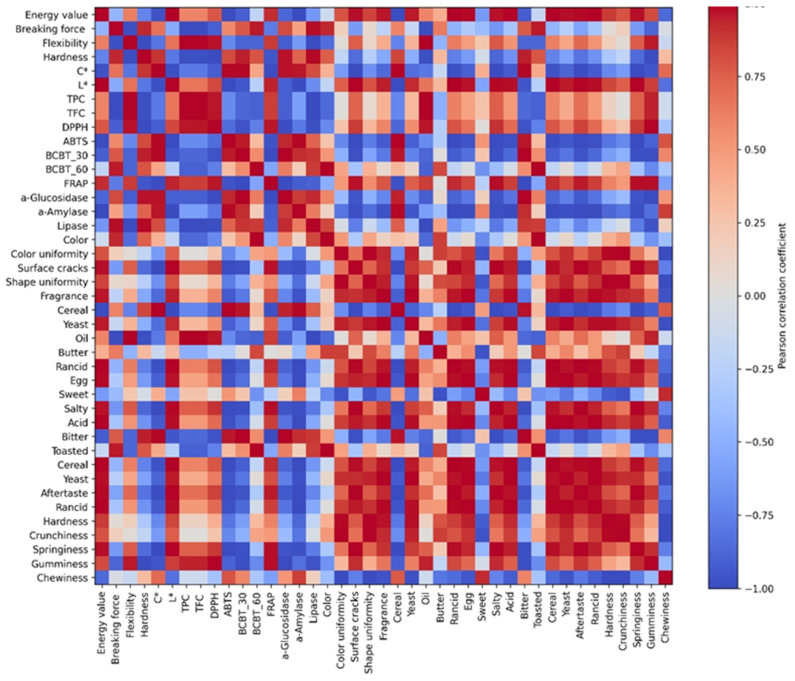
Heatmap of the Pearson’s correlation matrix among chemical, functional, textural and sensory attributes of the cookie formulations.

The Pearson’s correlation matrix (Figure 9) was used to explore associations among the chemical, functional, textural, and sensory attributes of the cookie formulations and to contextualise the patterns observed in the PCA. Positive correlations were identified among total phenolic content (TPC), total flavonoid content (TFC), and antioxidant capacity determined by the DPPH and FRAP assays, indicating a coherent alignment among these analytically related variables across the formulations.

Negative correlations were observed between phenolic-related parameters and several mechanical texture attributes, including hardness, breaking force, crunchiness, and chewiness. However, given the relatively limited quantitative variation among some variables, these relationships should be interpreted with caution and considered indicative of concurrent trends rather than evidence of direct or causal interactions. The observed associations likely reflect the combined influence of formulation composition on both functional and structural properties.

Sensory attributes such as bitterness, cereal notes, and aftertaste showed positive associations with phenolic content and antioxidant capacity, whereas sweetness and perceived chewiness were negatively correlated. These correlations suggest formulation-related shifts in sensory profiles; nevertheless, they should be regarded as descriptive tendencies rather than definitive determinants of sensory perception. In addition, correlations among colour parameters, toasting intensity, and texture attributes were detected, underscoring the interconnected effects of formulation and thermal processing on surface appearance and structural characteristics.

Overall, the correlation analysis highlighted interdependencies among functional, textural, and sensory variables and was consistent with the multivariate differentiation observed in the PCA. As an exploratory tool, however, the correlation analysis primarily serves to describe associative relationships and to support the formulation-driven grouping of samples, rather than to provide mechanistic interpretation.

## 4. Conclusions

This study presents an in vitro functional characterization of gluten-free and lactose-free cookies enriched with pomegranate seed flour (PSF), an underutilized by-product of juice processing, aimed at assessing its technological and biochemical potential as a food ingredient. PSF incorporation induced only minor changes in macronutrient composition while significantly increasing phenolic content, antioxidant capacity, and inhibitory activity against enzymes involved in carbohydrate and lipid digestion, with more pronounced effects at the highest enrichment level (10% PSF). All functional properties were evaluated exclusively through in vitro assays and should therefore be regarded as indicators of potential activity rather than evidence of physiological or clinical efficacy. Accordingly, no conclusions can be drawn regarding the prevention or management of metabolic syndrome, obesity, or related disorders, as bioaccessibility, bioavailability, and in vivo responses were not assessed.

Although both enriched biscuits improved the functional profile of the biscuit, the formulation with the addition of 5% proved to be an excellent compromise for functional improvement with improved technological and sensory characteristics compared with B10. Indeed, 5% PSF added to the biscuit showed a lower perception of bitterness and aftertaste, and hardness and breaking strength similar to sample B. Sample B10 maximizes the functional characteristics analysed, but an increase in sensory parameters is less required by consumers for biscuits.

Multivariate analysis revealed a clear dose-dependent differentiation among formulations, with PSF-enriched cookies, particularly those containing 10% PSF, closely associated with phenolic content and antioxidant-related variables, while control samples were mainly linked to conventional mechanical and sensory characteristics. Correlation analysis supported these trends, showing positive associations between phenolic compounds and antioxidant capacity, alongside negative correlations with selected texture parameters.

Although the in vitro extract concentrations exceeded those realistically achievable in the intestinal lumen following consumption of a typical serving, the present findings provide a proof of concept of the functional potential of pomegranate seed flour-enriched cookies. The observed antioxidant and enzyme inhibitory activities support the value of PSF as a promising upcycled ingredient for functional food design. However, the physiological relevance of these effects should be interpreted after further studies incorporating simulated gastrointestinal digestion and in vivo models, studies which are warranted by the need to better define bioaccessible fractions and achievable intestinal concentrations.

Overall, this work contributes to the sustainable valorisation of pomegranate by-products and highlights PSF as a promising ingredient for gluten-free bakery applications from a technological and in vitro functional perspective. Nevertheless, further investigations, including digestion models and bioavailability assessments, are required to clarify the nutritional relevance and potential health implications of PSF-enriched foods.

## Figures and Tables

**Table 1:** Cookie’s ingredients.

	E (Unit)	EVOO (g)	PMJ (g)	LM + V + S (g)	AS (g)	OF	CF (g)	RC	PSF (g)
B	3	60	60	✓	270	300	90	150	0
B5	3	60	60	✓	270	285	90	150	15
B10	3	60	60	✓	270	270	90	150	30

E: Eggs; EVOO: Extra virgin olive oil cv. Ottobratica; LM + V + S: Lemon peel + vanillin + a pinch of salt; AS: agave syrup; OF: Oat flour; RC: Coconut flour; CF: coconut flour; PMJ: Pomegranate juice; PSF: Pomegranate seed flour. ✓: added

**Table 2:** PSF constituents identified by LC-ESI/HRMS/MS analysis.

n	tR	[M−H]^−^	Mol Formula	[(M+FA)−H]^−^	Δppm	MS/MS	Name	Pomegranate
**1**	1.83	341.1079	C_12_H_22_O_11_		1.12	179.0550	Sucrose	X
**2**	2.35	353.0718	C_12_H_18_O_12_		1.01		Citric acid-*O*-hexoside	-----
**3**	2.80	191.0184	C_6_H_8_O_7_		−1.36	111.0073	Citric acid	X
**4**	4.47	399.1503	C_15_H_28_O_12_		1.50	353.1436	2-(hydroxy-propyl)-sucrose	X
**5**	5.24	205.0345	C_7_H_10_O_7_		1.22	143.0337, 111.0074	Methylcitric acid	-----
**6**	7.45	329.0876	C_14_H_18_O_9_		0.89	167.0338	Vanillic acid-*O*-hexoside	X
**7**	8.70	359.0989	C_15_H_20_O_10_		2.25	197.0445, 153.0544	Syringic acid-*O*-hexoside	-----
**8**	9.46	219.0502	C_8_H_12_O_7_		1.46	111.0073, 87.0073	Citric acid dimethyl ester	X
**9**	10.43	443.1918	C_21_H_32_O_10_		1.48	263.0153, 161.0444, 101.0229	Dihydrophaseic acid 3′-*O*-β-D-glucopyranoside	X
**10**	11.25	525.1612	C_24_H_30_O_13_		1.87	195.0693, 167.0338	Demethyloleuropein	X
**11**	11.25	785.0848	C_34_H_26_O_22_		2.01	300.9981, 275.0192, 249.0406	Pedunculagin II	X
**12**	11.64	799.0648	C_34_H_24_O_23_		2.87	300.9985, 273.0041, 247.0249	Granatin A	X
**13**	12.01	325.0926	C_15_H_18_O_8_		2.54	163.0889	Coumaric acid-*O*-hexoside	X
**14**	12.53	633.0735	C_27_H_22_O_18_		2.09	463.0521, 419.0613, 300.9986, 275.0195	Galloyl-HHDP-hexoside	X
**15**	13.35		C_19_H_30_O_8_	431.1919	0.72	205.1226	Roseoside	X
**16**	13.56	463.0518	C_20_H_16_O_13_		2.38	300.9985, 275.0199, 249.0403, 169.0131	Ellagic acid-*O*-hexoside	X
**17**	13.95	449.1089	C_21_H_22_O_11_		2.48	285.0557, 269.0450, 125.0231	Dihydrokaempferol-*O*-hexoside	X
**18**	14.86	415.1607	C_19_H_28_O_10_		2.01	269.0156, 107.1565	Icariside D1	X
**19**	14.99	951.0783	C_41_H_28_O_27_		1.48	300.9987, 463.0520, 273.0045, 247.0249, 166.9978	Granatin B	X
**20**	15.46	475.1806	C_21_H_32_O_12_		−0.78	205.0706, 101.0230	Kanokoside A	X
**21**	16.15	433.0410	C_19_H_14_O_12_		2.07	300.9904	Ellagic acid-*O*-pentoside	X
**22**	16.72	507.1504	C_24_H_28_O_12_		1.41	327.0869, 315.0866	Pomegralignan	X
**23**	16.81	300.9989	C_14_H_6_O_8_		3.34	284.0511, 229.0136	Ellagic acid	X
**24**	17.71	593.1523	C_27_H_30_O_15_		3.68	285.0404, 255.0298	Kaempferol 3-*O*-rutinoside	X
**25**	19.84	283.2640	C_18_H_36_O_2_		3.12	---	Stearic acid	------
**26**	26.87	271.0611	C_15_H_12_O_5_		2.80	151.0024	Naringenin	X
**27**	27.43	329.2333	C_18_H_34_O_5_		3.13	311.2217, 201.1125	Trihydroxyoctadecenoic acid (TriHOME)	-----
**28**	35.01	293.1753	C_17_H_26_O_4_		1.79		6-Gingerol	X
**29**	39.48	476.2775	C_23_H_44_O_7_NP		0.64	279.2329, 214.2105, 196.0375	l-PE (18:2)	-----
**30**	39.64	564.3293	C_26_H_50_O_7_NP		−0.43	504.3098, 279.2324, 224.0685	l-PC (18:2)	-----
**31**	40.16	315.2534	C_18_H_36_O_4_		1.87	187.1386, 83.0489	Di-hydroxy-stearic acid (DHSA)	-----
**32**	40.30	559.3116	C_28_H_48_O_11_		0.52	277.2166, 253.0919,	MGMG (C18:3)	-----
**33**	40.75	452.2777	C_21_H_44_O_7_NP		1.14	255.2324, 214.1928, 196.0370	l-PE (16:0)	-----
**34**	40.95		C_24_H_50_O_7_NP	540.3301	0.90	480.3076, 255.2323, 224.0684	l-PC (16:0)	-----
**35**	41.73	478.2930	C_23_H_46_O_7_NP		0.38	281.2479, 214.1916	l-PE (18:1)	-----
**36**	41.92		C_26_H_52_O_7_NP	566.3451	−1.17	506.3251, 281.2481, 224.0686	l-PC (18:1)	-----

‘X’ indicate presence, ‘-----’ indicate not identified.

**Table 3:** Macronutrient composition and energy value of PSF-enriched cookies.

Macronutrients	Sample			
	B	B5	B10	Sign.
Carbohydrates (g)	41.04 ± 5.87 ^a^	40.93 ± 4.92 ^ab^	40.81 ± 4.85 ^b^	*
Fats (g)	21.90 ± 0.43 ^a^	21.95 ± 0.48 ^a^	21.99 ± 0.56 ^a^	ns
Protein (g)	5.59 ± 0.86 ^a^	5.69 ± 0.90 ^a^	5.80 ± 0.94 ^a^	ns
Fibre (g)	3.35 ± 0.09 ^b^	4.55 ± 0.12 ^ab^	4.76 ± 0.18 ^a^	*
Energy value (kcal)	390.30 ± 8.34 ^b^	393.10 ± 8.43 ^b^	393.90 ± 8.62 ^a^	*
Energy value (kcal) units (~16 g)	62.40 ± 1.12 ^a^	62.90 ± 1.17 ^a^	63.00 ± 1.22 ^a^	ns
Energy value (kJ) units (~16 g)	261.10 ± 5.19 ^a^	263.20 ± 5.50 ^a^	263.70 ± 8.62 ^a^	ns

B: Control cookies; B5: Cookies enriched with 5% pomegranate seed flour; B10: Cookies enriched with 10% pomegranate seed flour. Differences between samples were evaluated by one-way ANOVA followed by Tukey’s test. Results followed by different letters are significantly different at * p < 0.05. ns: not significantly different.

**Table 4:** Textural parameters of pomegranate-enriched cookies.

Sample	Breaking Force (N)	Flexibility (mm)	Hardness (N)
B	35.37 ± 4.99 ^a^	1.06 ± 0.2 ^a^	43.42 ± 3.26 ^a^
B5	38.27 ± 4.92 ^a^	1.68 ± 0.1 ^a^	40.94 ± 5.63 ^a^
B10	21.72 ± 7.76 ^b^	2.09 ± 0.54 ^b^	31.23 ± 4.54 ^b^
Sign.	**	**	**

B: Control cookies; B5: Cookies enriched with 5% pomegranate seed flour; B10: Cookies enriched with 10% pomegranate seed flour. Differences between samples were evaluated by one-way ANOVA followed by Tukey’s test. Small letters within a column show significant differences as assessed by Tukey’s post hoc test. ** p < 0.01.

**Table 5:** In vitro antioxidant activity of pomegranate by-product-enriched cookies.

Sample	FRAP Test	β-Carotene Bleaching Test		DPPH Test	ABTS Test
		t = 30 min	t = 60 min		
	μM Fe (II)/g	IC_50_ (μg/mL)	IC_50_ (μg/mL)	Inhibition (%) at 1000 μg/mL	IC_50_ (μg/mL)
B	55.83 ± 3.91 ^b^	41.69 ± 5.80 ^c^	31.38 ± 3.43 ^b^	20.56 ± 2.85 ^c^	549.01 ± 11.46 ^b^
B5	64.32 ± 4.72 ^a^	20.68 ± 3.14 ^b^	14.57 ± 2.79 ^a^	23.62 ± 3.03 ^b^	443.05 ± 9.87 ^a^
B10	67.82 ± 4. 84 ^a^	16.03 ± 2.80 ^a^	12.72 ± 1.43 ^a^	32.04 ± 3.13 ^a^	406.53 ± 8.23 ^a^
Sign.	*	**	**	**	**

B: Control cookies; B5: Cookies enriched with 5% pomegranate seed flour; B10: Cookies enriched with 10% pomegranate seed flour. Data are shown as mean of triplicate and the experiment was independently replicated three times ± SD. Ascorbic acid, BHT and propyl gallate were used as positive controls in the antioxidant tests: ascorbic acid IC_50_ of 5.02 ± 0.79 μg/mL in the DPPH test, and 1.75 ± 0.12 μg/mL in ABTS test; BHT 63.26 ± 2.71 μMFe (II)/g in the FRAP test; propyl gallate was used in the β-carotene bleaching test with IC_50_ of 0.09 ± 0.04, and 0.08 ± 0.06 μg/mL at t = 30 and 60 min, respectively. Differences between samples were evaluated by one-way ANOVA followed by Tukey’s test. Results followed by different letters are significantly different at ** p < 0.01, * p < 0.05.

**Table 6:** Inhibition of α-amylase, α-glucosidase and pancreatic lipase by pomegranate by-product-enriched cookies (IC_50_, mg/mL).

Sample	α-Glucosidase	α-Amylase	Lipase
B	1.91 ± 0.09 ^a^	1.18 ± 0.06 ^b^	0.98 ± 0.11 ^b^
B5	1.26 ± 0.08 ^b^	0.37 ± 0.05 ^a^	0.97 ± 0.07 ^b^
B10	0.36 ± 0.04 ^a^	0.25 ± 0.07 ^a^	0.79 ± 0.05 ^a^
Sign	*	*	**

B: Control cookies; B5: Cookies enriched with 5% pomegranate seed flour; B10: Cookies enriched with 10% pomegranate seed flour. Data are shown as mean of triplicate and the experiment was independently replicated three times ± SD. Positive control: acarbose IC_50_ of 35.51 ± 1.10 μg/mL in α-glucosidase test and IC_50_ of 50.12 ± 1.13 μg/mL in α-amylase test whereas orlistat was used as a positive control in lipase test (IC_50_ value of 37.4 ± 1.0 µg/mL). Differences between samples were evaluated by one-way ANOVA followed by Tukey’s test. Results followed by different letters are significantly different at ** p < 0.01, * p < 0.05.

## Data Availability

The original contributions presented in this study are included in the article/Supplementary Materials. Further inquiries can be directed to the corresponding author.

## Supplementary Materials

The following supporting information can be downloaded at https://www.mdpi.com/article/10.3390/foods15030457/s1, Table S1. Macronutrient content, Bioactive content, Antioxidant and Enzymatic Inhibitory activities of pomegranate seed flour (PSF). Figure S1. Score plot of the principal component analysis (PCA) of cookie formulations.
