# Supplementary material for: Comprehensive LC-ESI-HRMS/MS Profiling and Assessment of Texture, Predicted Glycaemic Index, Antioxidant Activity and Digestive Enzyme Inhibition of Gluten- and Lactose-Free Cookies Enriched with Pomegranate By-Products
Source: Foods. 2026 Jan 28;15(3):457. doi: 10.3390/foods15030457 (PMC12897374; doi:10.3390/foods15030457)
Supplement: Supplementary file 1 [file foods-15-00457-s001.zip › foods-4087052-supplementary.pdf]

Supplementary Materials

Table S1. Macronutrient content, Bioactive content, Antioxidant and Enzymatic Inhibitory activities of pomegranate seed flour (PSF).

<table>
<tr><td colspan="5">Macronutrient content (per 100 g flour)</td></tr>
<tr><td>Protein (g)</td><td colspan="2">Carbohydrates (g)</td><td>Total Fats (g)</td><td>Dietary Fiber (g)</td></tr>
<tr><td>4.67</td><td colspan="2">18.74</td><td>11.49</td><td>28.92</td></tr>
<tr><td colspan="5">PSF Bioactive content</td></tr>
<tr><td colspan="3">TPC
(mg quercetin equivalents (QE) per 100 g of dried weight)</td><td colspan="2">TFC
(mg gallic acid equivalents (GAE) per 100 g of dried weight)</td></tr>
<tr><td colspan="3">0.09</td><td colspan="2">0.05</td></tr>
<tr><td colspan="5">PSF Antioxidant activity</td></tr>
<tr><td>FRAP Test</td><td colspan="2">β-Carotene Bleaching Test</td><td>DPPH Test</td><td>ABTS Test</td></tr>
<tr><td></td><td>t = 30 min</td><td>t = 60 min</td><td></td><td></td></tr>
<tr><td>μM Fe (II)/g</td><td>Inhibition (%)
at 100 μg/mL</td><td>Inhibition (%)
at 100 μg/mL</td><td>Inhibition (%)
at 500 μg/mL</td><td>Inhibition (%)
at 500 μg/mL</td></tr>
<tr><td>8.30 ± 0.05</td><td>61.31 ± 8.03</td><td>63.88 ± 8.11</td><td>42.58 ± 6.21</td><td>58.99 ± 7.01</td></tr>
<tr><td colspan="5">PSF Enzymatic inhibitory activity</td></tr>
<tr><td>α-Glucosidase</td><td colspan="3">α-Amylase</td><td>Pancreatic Lipase</td></tr>
<tr><td>Inhibition (%)
at 1000 μg/mL</td><td colspan="3">Inhibition (%)
at 1000 μg/mL</td><td>Inhibition (%)
at 1000 μg/mL</td></tr>
<tr><td>57.25 ± 4.77</td><td colspan="3">87.38 ± 9.43</td><td>32.33 ± 4.11</td></tr>
</table>

PSF: Pomegranate seeds flour extract. Data are shown as mean of triplicate and the experiment was independently replicated three times ± SD. Ascorbic acid, BHT and propyl gallate were used as positive control in the antioxidant tests: ascorbic acid IC_{50} of 5.02 ± 0.79 μg/mL in the DPPH test, and 1.75 ± 0.12 μg/mL in ABTS test; BHT 63.26 ± 2.71 μMFe (II)/g in the FRAP test; propyl gallate was used in the β-carotene bleaching test with IC_{50} of 0.09 ± 0.04, and 0.08 ± 0.06 μg/mL at t = 30 and 60 min, respectively. Acarbose IC_{50} of 35.51 ± 1.10 μg/mL in α-glucosidase test and IC_{50} of 50.12 ± 1.13 μg/mL in α-amylase test. Orlistat was used as a positive control in lipase test (IC_{50} value of 37.4 ± 1.0 μg/mL).

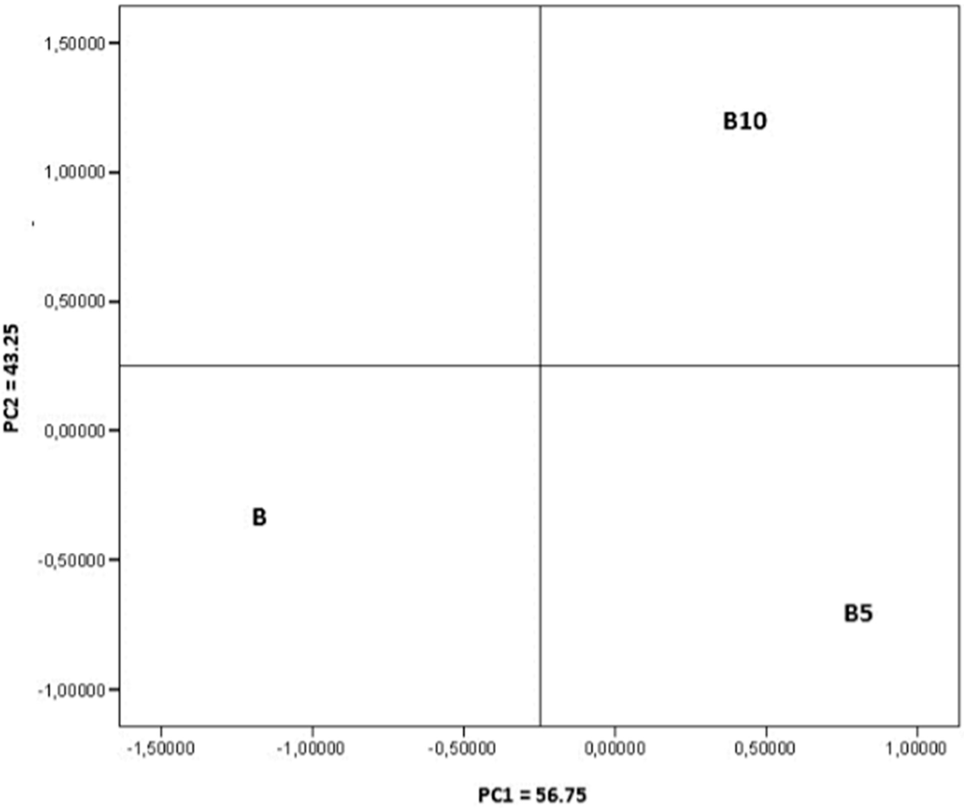

Figure S1. Score plot of the Principal Component Analysis (PCA) of cookie formulations.
